# A Rare and Unusual Presentation of Neurofibromatosis Type 1: Using Available Tools To Distinguish Neurofibromas From Mimicking Pathologies on CT Scan and MRI

**DOI:** 10.7759/cureus.39013

**Published:** 2023-05-14

**Authors:** Sammy Droubi, Keinan Taja, Amy Culler

**Affiliations:** 1 Radiology, University of Toledo, Toledo, USA

**Keywords:** neurocutaneous syndromes, phakomatoses, infected neurofibroma, plexiform neurofibroma, neurofibromatosis type 1 (nf-1)

## Abstract

Neurofibromatosis type 1 (NF-1) is the most common neurocutaneous syndrome. Despite its more common appearance relative to other phakomatoses, it has a large variety of disease manifestations that can, at times, make swift diagnosis more challenging if not readily recognized, especially when presenting in an atypical manner. Our case reveals an unusual presentation of NF-1. After initially presenting with a bug bite on the lip with progressive swelling and surrounding inflammatory changes despite treatment with oral antibiotics, a CT scan was performed and demonstrated inflammatory changes surrounding the lip with an adjacent inflammatory mass lesion. Due to hypoattenuating lesions within the retropharyngeal space and misinterpretation by the otorhinolaryngologist, aspiration was attempted but unsuccessful, and the patient’s condition worsened. Subsequent MRI was able to confirm the presence of numerous neurofibromas. The patient gradually improved on an extended course of antibiotics and was discharged in stable condition. Familiarizing oneself with the more specific imaging characteristics of this relatively common neurocutaneous disorder can help prevent incorrect or delayed diagnosis and ensure proper management. Furthermore, identifying these features on CT scan and MRI can differentiate them from other mimicking pathologies on each modality. Recognition of a scarcely reported infected neurofibroma as an established diagnostic entity could be important to include in the differential of similar cases in the future and subsequently aid in proper diagnosis and management.

## Introduction

Neurofibromatosis type 1 (NF-1) is a multi-systemic disease and is the most common of the neurocutaneous disorders affecting approximately one in every 2500-3000 individuals [1]. The most common features seen at diagnosis include cutaneous manifestations such as café au lait spots and axillary/inguinal freckling and neurological manifestations such as neurofibromas and gliomas [2]. However, a large spectrum of disease manifestations emphasizes the importance of identifying the more typical characteristics when present. Notably, approximately 30% of individuals with a neurofibroma will have NF-1. The presence of multiple neurofibromas or plexiform neurofibroma is pathognomonic for the disease [3]. As demonstrated in this case, recognizing the cardinal imaging features of NF-1, specifically neurofibromas, is crucial for preventing misdiagnosis and ensuring proper management.

## Case presentation

**Figure 1:**
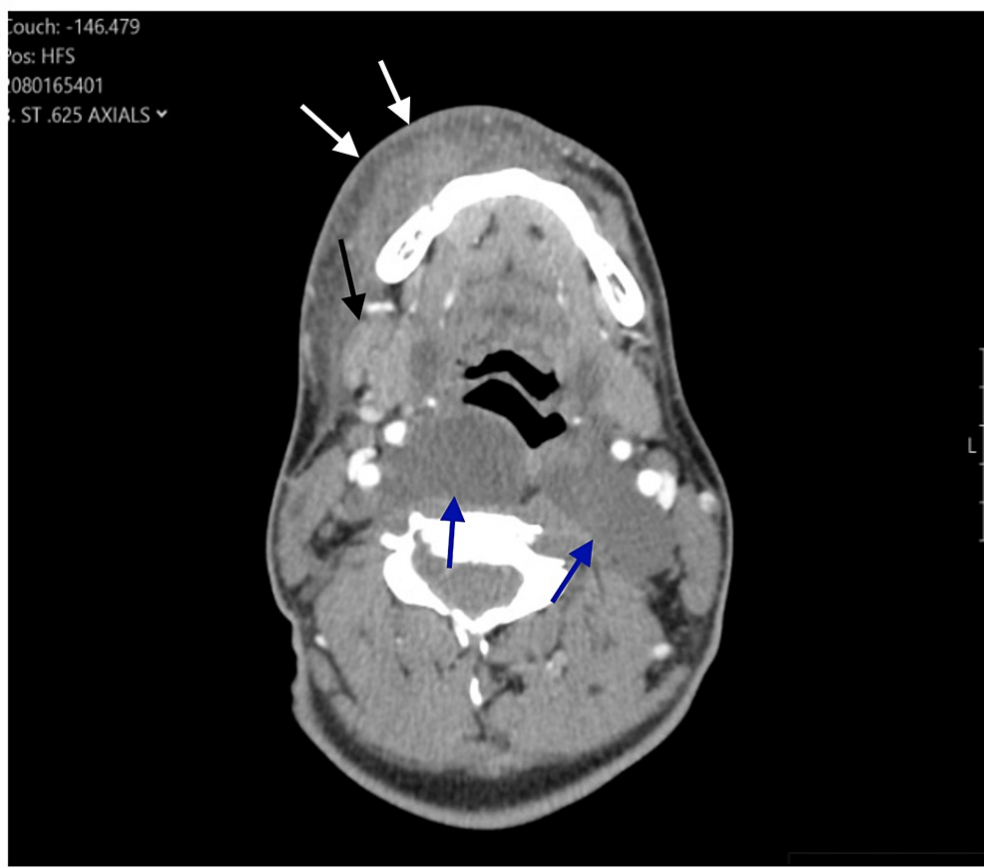
CT scan of the neck's soft tissues with contrast. White arrows: Skin thickening, inflammatory changes, and edema along the subcutaneous tissue of the right lower lip. Black arrow: Homogenous mass adjacent to the right submandibular gland with surrounding inflammatory changes. Blue arrows: Homogenous hypoattenuating retropharyngeal lesions, typical CT appearance of plexiform neurofibromas.

A 17-year-old male with a past medical history significant for developmental delay presented to the ER with facial swelling following a bug bite on his lower right lip five days earlier. The patient had a family history of neurofibromatosis, but a workup had not been completed. Vital signs included a fever of 100.9º F, a heart rate of 92 beats per minute, and a blood pressure of 110/72 mmHg. Physical examination revealed right lower swelling, erythema, and induration. His initial workup included a blood cell count and blood cultures. WBC count was 14.9 103/uL (normal: 4.00-10.60). RBCs, hemoglobin, and platelets were within normal limits. Serial blood cultures yielded no growth. The patient was immediately started on IV piperacillin-tazobactam 3.375 grams every six hours and IV vancomycin 500 milligrams every six hours. A CT scan of the neck's soft tissues with contrast was also obtained (Figure 1). It revealed thickening of the skin on the right lower lip, accompanied by significant soft tissue edema. Furthermore, stranding and heterogeneity were observed deep in this area, along the right mandible. Additional low attenuation mass-like lesions are seen within the parapharyngeal soft tissues extending along the carotid spaces bilaterally, within the retropharyngeal space, within the axilla bilaterally, and within the posterior neck musculature suspicious for plexiform neurofibromas.

**Figure 2:**
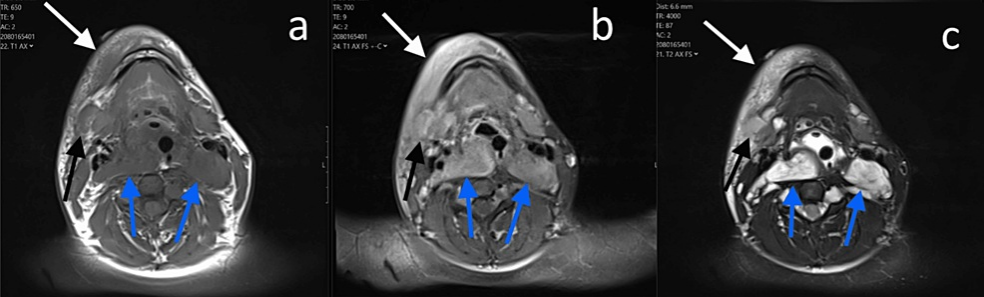
MRI of neck's soft tissues T1-weighted (a), T1-weighted postcontrast (b), and T2-weighted (c) sequences. White arrows: extensive edema and enhancement of the subcutaneous tissues along the right lower lip. Black arrows: T1 hypointense and T2 hyperintense mass demonstrating patchy hyperenhancement and surrounding inflammatory changes. Blue arrows: Large retropharyngeal masses demonstrating T1 hypointensity, patchy enhancement, and heterogeneous T2 hyperintensity, typical of plexiform neurofibromas.

Though no definite fluid collections were noted on the CT scan, the presence of inflammatory changes and mass-like heterogeneity near the right lower lip as well as adjacent retropharyngeal hypoattenuating lesions, was concerning to the ear, nose, and throat (ENT) physician. This, in addition to the history and clinical findings, prompted the ENT to proceed quickly with intervention for intended abscess drainage. However, the ENT failed to aspirate fluid, and the patient was subsequently transferred to the pediatric intensive care unit (PICU). He remained intubated for several hours due to continued concern for airway compromise. Subsequently, the day after admission, an MRI of neck soft tissues with and without contrast was ordered for further characterization of findings (Figure 2). This again demonstrated these masses throughout the soft tissues of the neck, axilla, as well as cervical and upper thoracic spine extending out of widened neural foramina. They demonstrated peripheral T2 hyperintensity with more central hypointensity and patchy postcontrast enhancement, consistent with plexiform neurofibromas. The extensive soft tissue edema and inflammatory changes along the right mandible were consistent with cellulitis. Within this region, there was a mass lesion demonstrating characteristics suggestive of likely an enlarged level 1b lymph node or possibly an inflammatory mass such as an infected neurofibroma. 

The patient was extubated later on day 1 following admission. He continued to improve by day 2, and was maintained on IV antibiotics. The patient was then switched to a once-per-day oral course of sulfamethoxazole-trimethoprim 800-160 milligram tablets upon discharge, resulting in a complete resolution of symptoms.

## Discussion

The most typical CT appearance of neurofibromas includes well-defined, non-enhancing hypoattenuating lesions (blue arrows in Figure 1). Depending on the histological composition, these may demonstrate mild enhancement, soft tissue density, and even calcification, but the vast majority do not. Plexiform neurofibromas, a subtype of neurofibroma commonly associated with NF-1, can have a similar appearance on a CT scan but are often more extensive and infiltrative. These features can mimic multiple etiologies, including adenopathy, sarcoidosis, metastatic disease, lymphangiomatosis, and abscesses [4]. Coincidental presentation of an NF-1 patient with surrounding soft tissue infection around hypoattenuating lesions, such as in our case, can further confound the clinical context and raise suspicion for other aforementioned pathologies, such as adenopathy and/or abscesses. In this setting, a more comprehensive discussion with the clinician and the recommendation of additional imaging, such as MRI, would be beneficial. MRI better distinguishes nerve sheath tumors from other entities and is quite useful for more complex cases such as this one. Neurofibromas tend to demonstrate low-to-intermediate T1 shortening, patchy enhancement, and heterogenous T2 hyperintensity (blue arrows in Figure 2). Plexiform neurofibromas tend to have a more characteristic appearance on T2-weighted sequences with peripheral hyperintensity and central hypointensity, often referred to as target signs [5,6]. Transformation to a malignant peripheral nerve sheath tumor is a feared complication of plexiform neurofibromas, usually demonstrating the increased size and T1 heterogeneity relative to benign neurofibromas. However, this can be difficult to differentiate. Target sign has been shown to be a reliable indicator favoring benignity [5]. Overlapping MRI features also exist between lymph nodes and neurofibromas, with relatively increased T2 hyperintensity and heterogeneity of neurofibromas often the best differentiating factor. To a lesser extent, overlapping features of neurofibromas and venous malformations have also been reported [6]. The MRI in our case revealed numerous masses with the characteristic target sign suggestive of plexiform neurofibromas. However, inflammatory changes surrounding the mass adjacent to the right submandibular gland (black arrows in Figures 1-2) demonstrated relatively less distinctive T2 characteristics and possible CT hyperdensity, making it difficult to confirm it as a neurofibroma, a reactive lymph node, or less likely a malignant peripheral nerve sheath tumor. The literature review did reveal a reported case of a patient with an infected neurofibroma who improved clinically with a 10-day course of antibiotics [7]. This could explain our patient's presentation, imaging characteristics, and recovery with prolonged antibiotics, but a biopsy was not done, and this could not be confirmed. Although the above-mentioned case report of an infected neurofibroma demonstrated findings of intralesional abscesses more suggestive of infection, there is still a scarcity of literature and a need for further research into imaging characteristics of neurofibroma infection, as it may be insufficiently acknowledged and treated [7].

## Conclusions

Our case stresses the importance of familiarizing oneself with the characteristics of this relatively common neurocutaneous disorder in order to prevent misdiagnosis and ensure proper management, even if manifesting with an atypical presentation. CT scans and MRI can illustrate these characteristics and, if properly recognized, can differentiate them from pathologies that mimic their features on each respective modality. Furthermore, we propose a scarcely reported entity of an infected neurofibroma as a potential cause of the patient's presentation. This emphasizes the importance of further research on this etiology so that it can be included in differentials and allow more prompt diagnosis and management when necessary.
